# Expression dynamics and relations with nearby genes of rat transposable elements across 11 organs, 4 developmental stages and both sexes

**DOI:** 10.1186/s12864-017-4078-7

**Published:** 2017-08-29

**Authors:** Yongcheng Dong, Ziyan Huang, Qifan Kuang, Zhining Wen, Zhibin Liu, Yizhou Li, Yi Yang, Menglong Li

**Affiliations:** 1 College of Life Science, Sichuan University, Chengdu, 610064 China; 2 College of Chemistry, Sichuan University, Chengdu, 610064 China

**Keywords:** Transposable elements, Rats, Expression patterns, Correlation, Organ, Sex, Age

## Abstract

**Background:**

TEs pervade mammalian genomes. However, compared with mice, fewer studies have focused on the TE expression patterns in rat, particularly the comparisons across different organs, developmental stages and sexes. In addition, TEs can influence the expression of nearby genes. The temporal and spatial influences of TEs remain unclear yet.

**Results:**

To evaluate the TEs transcription patterns, we profiled their transcript levels in 11 organs for both sexes across four developmental stages of rat. The results show that most short interspersed elements (SINEs) are commonly expressed in all conditions, which are also the major TE types with commonly expression patterns. In contrast, long terminal repeats (LTRs) are more likely to exhibit specific expression patterns. The expression tendency of TEs and genes are similar in most cases. For example, few specific genes and TEs are in the liver, muscle and heart. However, TEs perform superior over genes on classing organ, which imply their higher organ specificity than genes. By associating the TEs with the closest genes in genome, we find their expression levels are correlated, independent of their distance in some cases.

**Conclusions:**

TEs sex-dependently associate with nearest genes. A gene would be associated with more than one TE. Our works can help to functionally annotate the genome and further understand the role of TEs in gene regulation.

**Electronic supplementary material:**

The online version of this article (10.1186/s12864-017-4078-7) contains supplementary material, which is available to authorized users.

## Background

Rats and mice have been the most widely used models in biomedical research and drug development for many years [1–3]. However, a shift has taken place that mice rapidly overtake rats as the major model of choice [4]. As a result, the proportion of publications using mice models has increased from about 20% in the 1970s and 1980s to over 50% in the recent neuroscience-related researches. This shift might result from genome knockout technique, which was first used in mice, rather than in rats [5]. However, the rat is the preferred animal model for physiology, toxicology, nutrition, behavior and neoplasia studies. In addition, the rat can reduce the spread of drugs following intracranial injections [6]. These lead to urgent demands to study gene regulation patterns in rat. Benefiting from the creation and evolution of Rat Genome Database (RGD) [7] and the completion of the rat genome sequence in 2004 [8], we could look deep into the genetic rat models.

Transposable elements (TEs) were first discovered in maize and described as “controlling elements” of nearby genes [9]. At present, TEs have been found to exist in almost all species, with the proportions varying from ~1% in *Fusarium graminearum* to ~85% in maize genome [10–12]. It could be categorized into retrotransposons and DNA transposons. The former could be amplified through a copy-and-paste mechanism with an intermediate of the element-encoded RNA, while the latter utilizes a cut-and-paste mechanism to self-propagate with the intermediate of DNA [13, 14]. Retrotransposons could be further subdivided into long terminal repeats (LTRs), long interspersed elements (LINEs) and short interspersed elements (SINEs). L1 elements are the main retrotransposons in mammalian genomes with important roles in mutagenesis [15] and early cancer diagnosis [16, 17]. The new active TE integrations are usually removed from the population by purifying selection, while the high levels of methylation would buffer this effect and allow further adaptation and functionalization [18, 19]. TEs could function as transcription factor binding sites (TFBSs), enhancers, alternative promoters, cryptic splice sites and polyadenylation signals, insulators or modulate RNA abundance and shape RNA-protein regulatory network [20–24]. Particularly, as the enhancers, TEs could lead to a new group of genes to be expressed together and accelerate the formation of complex new pathways and functions [25].

Previous researches have suggested that some TE subfamilies may be transcriptionally activated following different tissues or environmental stress. For example, a subset of maize TE families can be activated in response to abiotic stress, including cold, heat, high salt or UV stress [26]. The expression of TEs in *Drosophila melanogaster* shows stage-specificity across 27 different developmental stages [27], especially *TART-B*, *copia* element and *Tom1*. In addition, it was also documented that several individual TEs could influence the expression of nearby genes [28–30]. Faulkner et al. [31] firstly demonstrated that TEs are the integral part of the transcriptome and their transcripts are generally tissue specific and could influence the transcriptional output of the human and mouse genome. The rice DNA transposon *mPing* resulted in up-regulation of nearby gene in response to cold or salt stress [32]. Lynch et al. demonstrated that the ancient TEs could donate *cis*-regulatory elements to recruited genes, especially for human decidual stromal cells, in which 194 ancient TEs were enriched within *cis*-regulatory elements [33]. Many reports illustrated that some TEs are tissue-specific and could influence the expression of nearby genes, however its influence range and time course remains unclear.

In this paper, we focused on the expression patterns of TEs and their relations with the closest genes in different organs, sexes and ages of rat by using the RNA-seq data [34]. The traditional methods considering only uniquely mapped reads would lead to underestimate expression signal of TEs, because TEs usually have high copy numbers. In this study, we adopted the *iteres* tool to estimate the expression levels of TE subfamilies for its ability of dealing with non-unique mapped reads [35].

The work of this study could be divided into two main themes. In the first section, the TEs spread throughout the whole genome and this distribution raises some interesting questions—whether the TE subfamilies expression are organ-, age- and sex-dependent. If so, what’s the pattern? We examined the expression profiles of TEs and found the fraction of differentially expressed TEs (DETEs) varied greatly among organs, developmental stages and sexes. Most SINEs, which were commonly expressed in all conditions, were the major TE types with commonly expression patterns. In contrast, LTRs were more likely to appear specifically expression patterns. In the second theme, the Pearson correlation coefficient (PCC) of expression signals between individual TE and its nearest gene was estimated. In some cases, the PCC was independent on the distance between TEs and the nearest genes. Some LTRs sex-dependently associated with their nearest genes.

## Methods

### TEs distribution in various genomic compartments

To examine whether TEs have tendency to spread in a specific genomic compartment, we estimated TEs distributions in CDS exon, UTR exon, Intron and Intergenic regions. For this, we used the intersect tool from the BEDTools package v2.26.0 [36] and required a minimal overlap fraction of 50%. When a TE was located in multi genomic compartments, it was then assigned to the compartments according to the following priority: CDS exons > UTR exons > Introns > Intergenic regions [37]. For example, if a TE region was overlapped with both UTR exon and Intron, it would be assigned to UTR exons.

### Data sources and data processing

RNA-seq data sets for the rat were obtained from [34]. A total of 320 samples consist of 11 organs: Adrenal gland (Ad), Brain (Br), Heart (He), Kidney (Ki), Liver (Li), Lung (Lu), Muscle (Mu), Spleen (Sp), Thymus (Th), Testis (Te) and Uterus (Ut). Each organ was studied in four developmental stages: 2-week-old, 6-week-old, 21-week-old and 104-week-old. Except for Te and Ut, both sexes were studied for each organ. There were four biological replicates in specific organ, age and sex. According to the above description, there are 9 (organ) × 4 (age) × 2 (sex) × 4 (biological replicate) +2 (organ) × 4 (age) × 1 (sex) × 4 (biological replicate) = 320 samples.

**Fig. 1:**
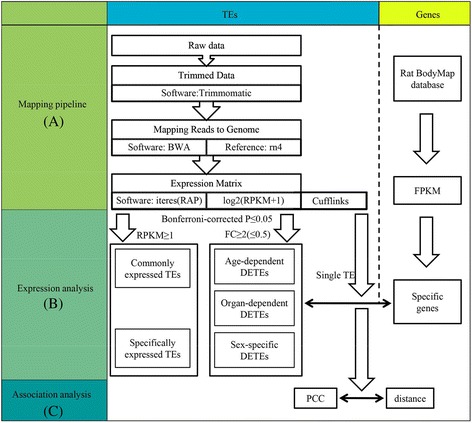
Procedure to calculate differently expressed TEs and determine the relations between genes and TEs. **a** Mapping pipeline. Trimmomatic was used to trim data, and then BWA v0.7.12 with default parameters was used to mapping reads to genome. The iteres was used to calculate RPKM of all TE subfamilies in all samples. The cufflinks was used to calculate RPKM of each TE in all sample. The expression value of genes was downloaded from Rat BodyMap database. **b** Fold change and Bonferroni-corrected *p*-value were used to evaluate DETEs. **c** We calculated PCC and distance between TE and its nearest gene, and then explored relation between PCC and distance

All annotation and genome information were obtained from UCSC Genome Brower (rn4). Repeats classified as low complexity, simple repeat, satellite, scRNA or unknown were discarded and only SINEs, LINEs, LTRs and DNA transposons were retained. Reads were first trimmed using Trimmomatic [38], then mapped by BWA v0.7.12 [39]. In order to make full use of high quality sequencing reads, Reads Per Kilobase of exon model per Million mapped reads (RPKM) was calculated for subfamily by *iteres*, which is developed and maintained based on the Repeat Analysis Pipeline (RAP) [35]. In addition, RPKM was also calculated for single TE by cufflinks v2.2.1 [40]. Lastly, RPKM with adding 1 was transform by log2 (Fig. 1a).

In this study, TE subfamilies were considered to be expressed with the averaged RPKM ≥ 1. A TE subfamily was defined as “commonly expressed TEs” if it was expressed in all organs, developmental stages and sexes. Circos [41] was used to draw the graph of the number of DETEs among organs and links between organs and classes. The clustering of TE subfamilies was performed using Average linkage in MATLAB. Principal variance component analysis (PVCA) leverages the strengths of principal components analysis and variance components analysis to quantify the corresponding proportion of variation of each effect [42]. In this study, it was used to quantify the relative contributions of effects (organ, age, sex and replicate) to total model variance based on the expression matrix of TE subfamilies in different samples.

### Identification of differentially expressed and organ-enriched TE subfamilies

In order to compare with expression of genes, we adopted same methods with Yu et al. [34] to identify enriched TEs. For the sake of completeness, we would describe these methods in brief.

A TE subfamily was defined as the DETE between two organs if t-test with a Bonferroni-corrected *P*-value was ≤ 0.05 and fold change (FC) was ≥ 2 (overexpressed) or ≤ 0.5 (underexpressed). The intersection of DETEs that were overexpressed in any other 10 organs were defined as organ-enriched TE subfamilies. The development-dependent DETEs were evaluated by comparing different developmental stages for each organ. The condition was FC ≥2 or ≤0.5 plus Bonferroni-corrected *P*-value ≤ 0.05. Except for testis and uterus samples, other 288 samples were separated into 36 groups according to the organ types and developmental stages. FC and t-test were also performed between male and female to identify sex-dependent DETEs in each group.

In each organ, the FC was calculated between two adjacent developmental stages, with the older developmental stage as numerator, in other words, 104- versus 21-weeks old, 21- versus 6-weeks old and 6- versus 2-weeks old.

A TE subfamily with FC ≥2 or ≤0.5 plus Bonferroni-corrected *P*-value ≤ 0.05 was divided into the “up” pattern or “decrease” pattern, respectively. The other TE subfamilies were divided into “maintain”. Therefore, a TE subfamily could be divided into 1 out of 27 patterns in each organ, ranging from up-up-up (UUU), maintain-maintain-maintain (MMM), to decrease-decrease-decrease (DDD).

## Results

The single-end RNA-seq data of 320 samples from Yu et al. [34] were employed to quantify the expression levels of the TE subfamily as well as the individual TE. In addition, we associated TEs with genes by using distance between TEs and transcriptional start sites (TSSs). A flowchart of the whole work was shown in Fig. 1.

### Proportion of TEs in various genomic compartments

**Fig. 2:**
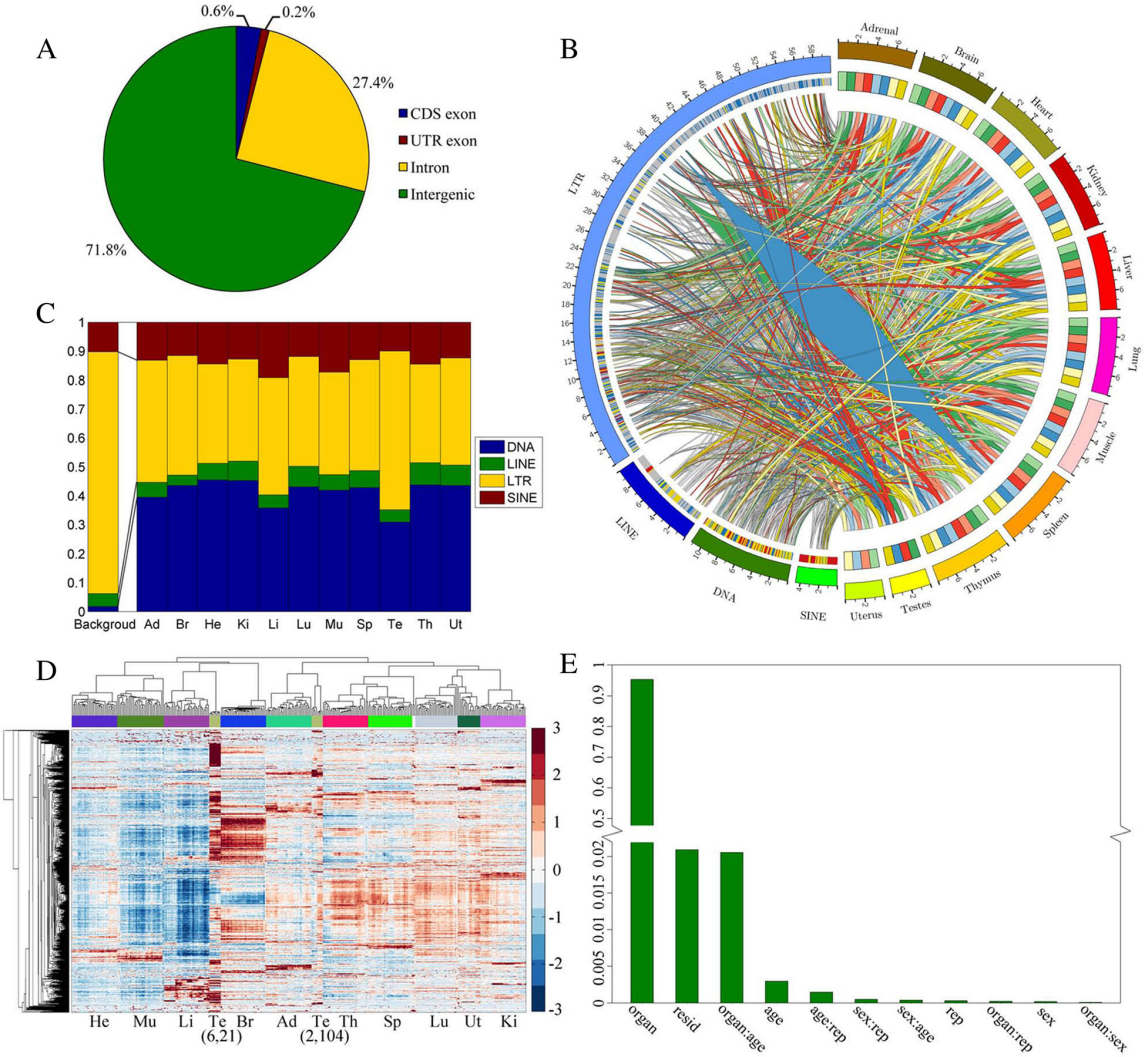
Overview of TEs in the genome and various conditions. **a** The proportion of all TEs in various genomic compartments. The allocation of TEs obeys this priority: CDS exons > UTR exons > Introns > Intergenic regions. **b** The expression summary of TE subfamilies in all groups. This panel can be divided into the left and right sides. Right sides from outside to inside represent organ and four developmental stages in both sexes. Green represents 2-week-old rats. Red represents 6-week-old rats. Blue represents 21-week-old rats. Yellow represents 104-week-old rats. Light color represents male and dark color represents female. So, right sides contain 80 groups. Left sides from outside to inside represent TE class and relation with organs, developmental stages and sexes. In internal layer, red represents commonly expressed TEs; gray represents zeros expressed TEs; yellow represents hub TEs and blue represents non-hub TEs. In order to visualize, the relations between commonly expressed TEs and organs were not drawn. A line represents that a TEs expressed in a group. **c** The proportion of subfamily types that simply expressed in each TE class in 11 organs. For comparison, “Background” TEs represent those simply found in the genome. **d** Hierarchical cluster analysis of TE subfamily expression signals across 320 samples. The row represents TEs, and the column represents samples. **e** The relative contribution was calculated by PVCA, including main effects (organ, age, sex and replicate) and their combinations

Analysis was performed firstly on the genomic locations for the TEs records in the UCSC (Fig. 2a). Most TEs were located in intergenic region and intron, and only 0.8% TEs in exon of gene, which was consistent with the related reports [43, 44]. Different TE classes also exhibited significant location trends in genomic compartments (Additional file 1: Table S1). SINEs tended to locate in gene-rich regions, which might result from the bias insertion of SINEs [13]. LTRs and LINEs tended to locate in intergenic regions.

### Quantify and evaluate expression signal of TE subfamilies

The Mammalian TEs are hierarchically divided into classes, families and subfamilies. The following analysis mainly focused on four major classes LINE, SINE, LTR and DNA consisting of 56 families and 855 subfamilies in rat. We used BWA V0.7.12 [39] and iteres [35] to map reads and quantify the expression levels of TE subfamilies (Fig. 1a). The final expression matrix, consisting of 855 subfamilies across 320 samples, was got for the further analysis. After normalization, the pair-wise PCC was calculated between the TE subfamilies expression levels for any two of the four biological replicates (Additional file 2). High reproducibility was detected for each sample group, with PCC values from 0.9228 to 0.9847 and the standard error from 0.0013 to 0.0412.

The general expression patterns (RPKM ≥ 1) of TEs were shown (Fig. 2b) for each sex at each time point in each organ. 462 subfamilies were expressed in at least one among 80 groups with 14,666 links as shown in Fig. 2b. This indicated that some TE subfamilies might specifically express in partially conditions. The rest of 393 subfamilies, termed zero expressed TEs, were not expressed in any groups. In retrotransposons, SINEs followed by LINEs and LTRs, exhibited high occurrence frequency. In LINEs, partial L1 showed significant activity. These were in accordance with the reports [45–48]. Sixty-six of 462 subfamilies were defined as “commonly expressed TEs” since they were expressed in all 80 groups (Additional file 1: Table S2 and S7). Interestingly, most SINEs (68.89%) were commonly expressed TEs (Table 1), which was also the major TE class with commonly expression patterns (46.97%). It has been demonstrated that SINE elements could function as the main TE-derived TFs binding sites to regulate gene expression in mouse [49]. It was also confirmed that commonly expressed *PB1D9* element belonging to SINE is a strong promoter in rat [50]. In addition, a few LTR elements, stimulating to transcriptional initiation [21, 51, 52], were also commonly expressed TEs, such as ERV repeat elements. Additionally, the DNA element *MER20* was found as a commonly expressed TE. It has been demonstrated that *MER20* associated with enhancers, repressors and histone modifications [53]. And this TE was also high conservation and regulatory potential. Consequently, we made analysis on the specifically expressed TEs. Here, the specifically expressed TEs were defined as those only expressed in male/female at a particular developmental stage in an organ, while the organ-specific TEs were those only expressed in an organ. Many LTRs were specifically expressed TEs (6.18%) or organ-specific TEs (13.52%) (Additional file 1: Tables S3, S4, S8 and S9). Similar observations were also reported that many LTRs could exhibit organ-specific expression pattern [52, 54], and exert organ-specific regulation on adjacent genes [55, 56]. Most zero expressed TEs (82.74%) were LTRs. LTRs, the descendants of exogenous retroviruses, could be integrated into the genome of germ cells. Most of which would gradually lose the function and exit the host cell. Those LTRs expand in their host genome by vertical transmission, but only act as retrotransposition and has no function for infection [57, 58]. Furthermore, most L1 (63.16%) was found as the zero expressed TEs. This has been demonstrated in a number of studies that many L1 elements are truncated at the 5′ end leading to lose some regulatory regions, especially TSS [59, 60]. This indicated that those L1 elements would not be competent of further retrotransposition and might become zeros expressed TEs.

**Table 1:** The distribution of commonly expressed TEs in four classes

Class	Totals	Common expression	Percentage 1(%)	Percentage 2(%)
SINE	45	31	68.89	46.97
LINE	100	4	4	6.06
DNA	112	19	16.96	28.79
LTR	599	12	2.00	18.18

The second column shows the number of each class. The third column shows the number of commonly expressed TEs in each class. The fourth column shows the proportion of commonly expressed TEs in each class. The last column shows the proportion of commonly expressed TEs in all commonly expressed TEs

We then investigated the effects of organ, age and sex on the TEs expression by using the PVCA (Fig. 2e). We found ~95% variance resulted from organs, while other effects had limited variance even less than the residual variance of the model. Here, it should be noted that the Y chromosome has not been sequenced for rat. This would lead to underestimate the effect from the sex. We therefore look deep into the organ related TE subfamily expression patterns. The results suggested similar TE expression patterns with genes. For example, for both TEs and genes, the highest expressed number was found in the lung and testis, while the lowest number in the liver, muscle and heart (Additional file 3). On average, in the subfamily level there were 184 (21.52%) TE subfamilies expressed in each organ. In the class level the proportion of the expressed TE classes were similar across 11 organs (Fig. 2c). A significant decrease was observed for the expressed LTR proportion against the background. The number of expressed LTRs and specifically expressed LTR subfamilies were the highest in the testis of all organs (Additional file 1: Table S9).

We then performed a hierarchical cluster analysis to obtain an overview of TE expression patterns across 320 samples. The clustering of TE expression profiles suggests that organ has a substantial effect on the transcriptome except for the testis (Fig. 2d). Compared with the clustering of gene expression profiles [34], the TE clustering performed better in organ discrimination, since one of the four developmental stages in thymus was classified as spleen by the former.

### The identification of DETEs

**Fig. 3:**
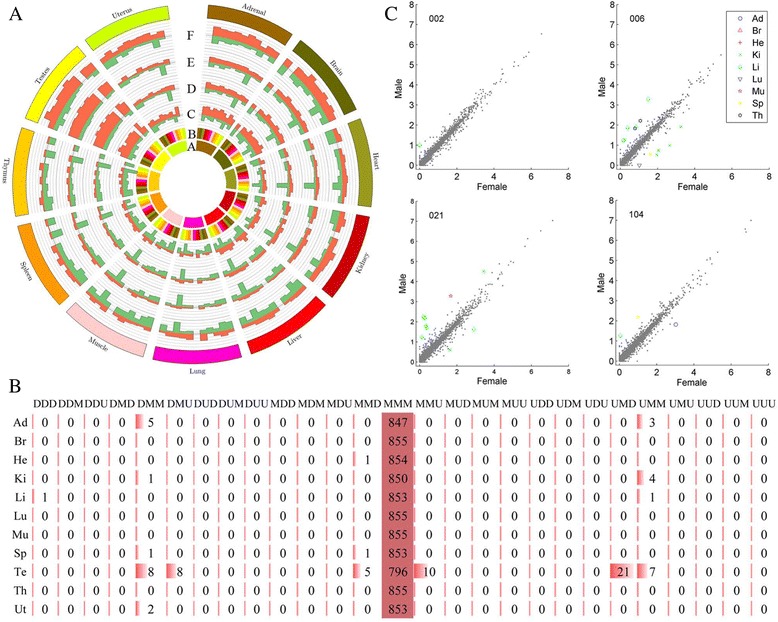
Patterns of DETEs. **a** Organ-enriched TE subfamilies. Shown is the relative number of DETEs between any two organs in four developmental stages. A represents organ under comparison; B represents the 11 organs being compared with organ A. C to F, respectively represent 2-, 6-, 21- and 104-week-old rats. Orange represents over expression and green represents underexpression. **b** The number of development-dependent TE subfamilies within each pattern of each organ. **c** Expression signal of four development stages from nine organs are depicted in the scatter plots. X-axis represents female. Y-axis represents male

DETEs were estimated as those with fold change (FC) ≥ 2 and Bonferroni-adjusted *P*-value ≤ 0.05 (Methods). The TE subfamily expression levels were compared between any two organs for the 4 developmental stages to identify organ-dependent DETEs (Fig. 3a). Most DETEs were observed in testis, adrenal and brain, while few in spleen, muscle and liver, which shared similar trends with genes. We then identified organ-enriched TE subfamilies. Only 10 TE subfamilies (1.17%) were enriched (Additional file 1: Table S10). In these organ-enriched subfamilies, 4 subfamilies (40%) were enriched in brain. Interestingly, 41.0% of these organ-enriched genes were reported in brain by [34].

The comparisons were performed between any two developmental stages in each organ to evaluate development-dependent DETEs. We identified 84 DETEs that appeared at least one of the 11 organs. The number of DETEs varied significantly among organs/developmental stages (Additional file 1: Table S5). When compared with the 2-week-old rats, a number of DETEs were detected in other developmental stages, which was similar with the reports by Yu et al. [34]. Among all organs, testis contained the most development-dependent DETEs. The inner comparison within young (6- and 21-week-old) and atrophying (2- and 104-week-old) testes showed a handful of DETEs. We also performed a time course analysis by comparing any two adjacent developmental stages to evaluate transcriptomic activities alterations through the life cycle of the rat (Methods). Each TE could be grouped into one of the 27 possible patterns. The number of subfamilies for each pattern in each organ was shown in Fig. 3b. MMM was the most frequently observed expression pattern, which indicated the stable expression level over the lifespan. In addition, DMM, UMM, MMD and MMU were also frequently observed.

We finally identified sex-dependent DETEs in each organ or developmental stage. Some TEs were differentially expressed between female and male rats, especially in kidney and liver (Additional file 4). Half of the sex-dependent DETEs were observed in 6-week-old rats and ~34.6% in 21-week-old rats (Fig. 3c; Additional file 1: Tables S6 and S11). This may result from adolescence and sexual maturity. Because of organs atrophy in aging rats or non-development in juvenile, there were only four DETEs in 104- and 2-week-old rats. 84.6% sex-dependent DETEs belonged to LTRs, and the others belonged to DNA. In other words, LTRs had sex-dependent expression, so we put forward a hypothesis that LTRs had sex-dependent association with nearby genes.

As aforementioned, organ-dependence, development-dependence and sex-dependence of DETEs exhibited consistent patterns with those of differentially expressed genes (DEGs). We therefore made a further investigation into the associations between genes and TEs.

### The relations between genes and TEs

More detailed, we evaluated the expression levels of single TEs by using BWA V0.7.12 [39] and cufflinks v2.2.1 [40], and then got the nearest gene of each TE by using the closest tool from the BEDTools package v2.26.0 [36]. Since the TEs may exert impacts on the expression of the proximal genes [61, 62], we calculated the PCC of the expression levels between the TE and its nearest gene, and then investigated whether the PCC would be related with their distance.

**Fig. 4:**
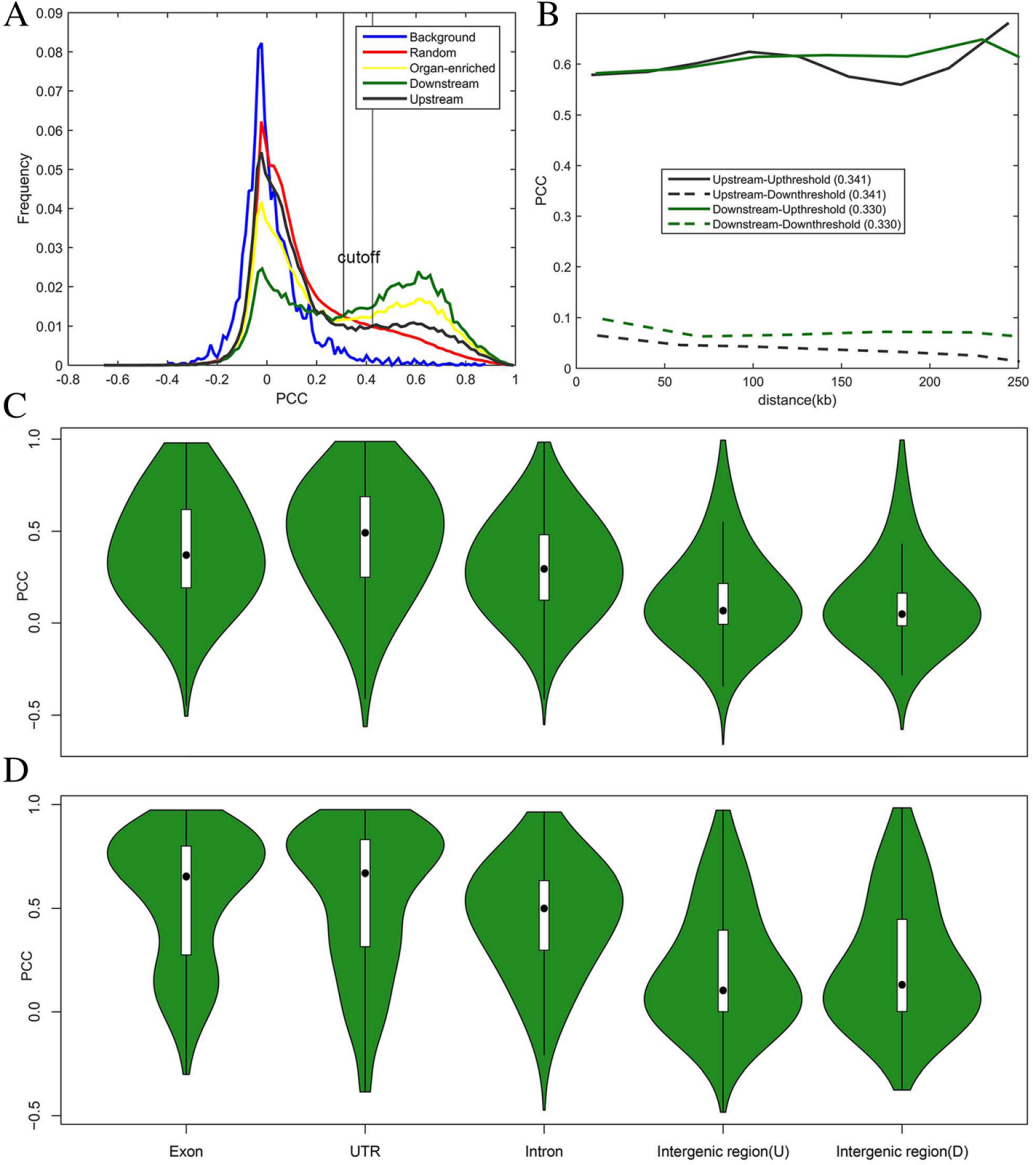
The relations between TEs and organ-enriched genes. **a** Frequency distribution map of PCC in organ-enriched gene-TE pairs. Organ-enriched gene-TE pairs above cutoff were identified as significant correlations. **b** The relation between PCC and distance. **c** Comparison of distributions of PCC about all gene-TE pairs in various genomic compartments. **d** Comparison of distributions of PCC about all organ-enriched gene-TE pairs in various genomic compartments. The distribution of PCC is shown using violin plot

As showed in Fig. 4a, significant bimodal distribution (p < <0.001) was observed for all gene-TE pairs against the background as the randomly selected gene-TE pairs. Those organ-enriched gene-TE pairs showed a peak around 0.62. The methods for gene-TE pairs selection were described in Additional file 5. We then categorized the TEs associated with organ-enriched genes into two groups that TEs were located in the upstream of TSSs (UTSSs) and downstream of TSSs (DTSSs). The latter showed more significant bimodal distribution than the former. When focusing on only the gene-TE pairs with significant correlations (Fig. 4a), we found their PCC didn’t depend on their distance (Fig. 4b). The mean distance for the DTSSs was 1.5 times larger than for the UTSSs. We further asked whether the PCC distributions would differ in genomic compartments, including CDS exons, UTR exons, introns and intergenic regions. Interestingly, both for all gene-TE pairs and the organ-enriched gene-TE pairs, the highest median PCC value was found between genes and UTR TEs, followed by exon, intron and intergenic TEs (Fig. 4c, d). As previously reported that TEs have been co-opted as tissue-specific enhancers and tissue-specific primary or alternative promoters, particularly the LTRs [62, 63].

The same process was performed on the sex-dependent DEG-TE pairs. Some sex-dependent DEGs were detected in multiple organs and ages. Interestingly, more significant correlation peaks could be detected for those sex-dependent DEGs shared by more organs/ages (Additional file 6). We focused on sex-dependent DEG-TE pairs in which the genes appeared in one or five times. The results showed that the latter appeared higher median and frequency (Additional file 7). It indicated that some TEs sex-dependently associated with proximal genes, and more than half of TEs were LTRs.

Development-dependent gene-TE pairs were similarly analyzed and similar results were obtained (Additional files 8 and 9). For the genes with UUU expression pattern, significant correlations were observed with their closest TEs.

## Discussion

We focused on both the expression of TEs and spatiotemporal influence of TEs on genes. We adopted iteres [35] to calculate expression levels of TEs in different samples due to the high copy number of TEs. Most commonly expressed TEs were identified as the SINEs and vice versa. It has been reported that Alu family elements, belong to SINEs, would be enriched nearby the housekeeping genes [64, 65], and the distribution of SINEs conserved across species [66]. Most specifically expressed TEs were LTRs. The phenomena might result from TE-derived TFs binding sites of SINEs for nearby genes and tissue-specific regulation of LTRs [21, 49, 51, 55]. Except for the significantly alternative expression level of LTR in testes, TE classes showed even expression levels among organs. The results of hierarchical cluster analysis showed that compared with genes, the expression of TEs could better represent differences between organs. We used PVCA to quantify the sources of variance about TEs expression in different organs, ages, sexes and biological replicates. The results suggested that the differences mainly resulted from organs. The reason for the low variance by sex might be that Y chromosome were not sequenced in this dataset. This was consistent with the following differential expression analysis. For example, compared with the amount of organ-dependent DETEs, only 18 subfamilies showed sex-dependent expression dominated by the LTRs. A hypothesis was then made that TEs may sex-dependent associate with nearby genes based on above findings. Our result support this hypothesis. DEGs and DETEs showed similar expression patterns, such as underexpressed in the liver, muscle and heart, and overexpressed in the testes and brain. Most sex-dependent DEGs and DETEs were found at 6 or 21 weeks. Few genes and TEs continuously changed (UUU, DDD) through the lifespan.

TEs are usually considered as the deleterious or the neutral element of genomes, but this effect can be buffered to allow further adaptation and functionalization [18, 26, 67]. The last results may lead to interplay between TEs and genes, which may have important functional contributions to tissues, ages or sexes. We linked individual TE to the nearest gene, then calculated the linear correlation of expression signals and the distance of gene-TE pairs. The results indicated that most TEs positive correlated with their nearby genes at the expression levels. In some cases, PCC didn’t depend on the distance between gene and its nearest gene.

## Conclusions

This study presented a comprehensive analysis on the TEs expression patterns from organ types, time course and sexes aspects. The results of our present study suggested that most SINEs, which were commonly expressed in all conditions, were the major TE types with commonly expression patterns. In contrast, LTRs were more likely to exhibit specific expression patterns. Most specifically expressed TEs were also LTRs. Similar expression patterns were shown between DEGs and DETEs. Furthermore, the temporal and spatial influences of TEs on genes were evaluated. The results indicated positive PCCs between most TEs and their nearby genes at the expression levels. In some cases, PCC didn’t depend on the distance between gene and its nearest gene.

In this paper, we used a pipeline to calculate expression level of TE subfamilies in different organs, ages and sexes. The pipeline could also be used in other conditions, such as biotic stress and environmental change. Our works could promote the understanding of the regulation model of rat.

## Additional files

Additional file 1: Table S1-S11. (PDF 49 kb)

Additional file 2: Figure S1. The distribution of pair-wise Pearson correlation coefficient (PCC) in each organ. X-axis represents 480 values in 11 organs, and Y-axis represents PCC between any two of the four biological replicates. Each color represents a kind of organ. Except for Te and Ut, other organs contain 48 dots. (TIFF 298 kb)

Additional file 3: Figure S2. The number of expressed TEs across 11 organs. The x-axis indicates organs and the y-axis indicates the number of expressed TEs. (TIFF 963 kb)

Additional file 4: Figure S3. Sex-specific DETEs. Nine organs from four developmental stages. All expression signals are depicted in the scatter plots. X-axis represents expression signal of female rats, and Y-axis represents expression signal of male rats. Non sex-specific TEs are grey color, while DETEs are colored. (TIFF 232 kb)

Additional file 5: Figure S8. The methods for gene-TE pairs selection. We got all gene-TE pairs by distance. In all genes, some genes were organ-enriched. In all TEs, some TEs were located in UTSSs, while some TEs located in DTSSs. We randomly selected genes and TEs, and these gene-TE pairs were used as Background. We randomly selected gene-TE pairs from all gene-TE pairs that were used as Random. TEs located in UTSSs and organ-enriched genes were combined as Upstream. TEs located in DTSSs and organ-enriched genes were combined as Downstream. (TIFF 639 kb)

Additional file 6: Figure S4. Frequency distribution map of PCC between TEs and sex-specific genes. (TIFF 384 kb)

Additional file 7: Figure S5. Violin plot of PCC between TE and sex-specific gene that appeared 5 times (A) and 1 time (B) in different organs and development stages. (TIFF 923 kb)

Additional file 8: Figure S6. Frequency distribution map of PCC between TEs and development-dependent genes. (TIFF 418 kb)

Additional file 9: Figure S7. Violin plot of PCC between TE and development-dependent gene that appeared 3 times U (A) and 1 time D (B). (TIFF 797 kb)

## Abbreviations

Ad: Adrenal gland
ADRs: Adverse drug reactions
Br: Brain
CDS: Coding sequence
DDD: Decrease-decrease-decrease
DEGs: Differentially expressed genes
DETEs: Differentially expressed TEs
DTSSs: Downstream of TSSs
FC: Fold change
He: Heart
Ki: Kidney
Li: Liver
LINEs: Long interspersed elements
LTRs: Long terminal repeats
Lu: Lung
MMM: Maintain-maintain-maintain
Mu: Muscle
PCC: Pearson correlation coefficient
PVCA: Principal variance component analysis
RAP: Repeat analysis pipeline
RPKM: Reads Per Kilobase of exon model per Million mapped reads
SINEs: Short interspersed elements
Sp: Spleen
Te: Testis
TEs: Transposable elements
TFBSs: Transcription factor binding sites
Th: Thymus
Ut: Uterus
UTSSs: Upstream of TSSs
UUU: Up-up-up
