# Supplementary material for: Expression dynamics and relations with nearby genes of rat transposable elements across 11 organs, 4 developmental stages and both sexes
Source: BMC Genomics. 2017 Aug 29;18:666. doi: 10.1186/s12864-017-4078-7 (PMC5576108; doi:10.1186/s12864-017-4078-7)
Supplement: Supplementary file 1 — (PDF 49 kb) [file 12864_2017_4078_MOESM1_ESM.pdf]

S1 Table: The differences of TEs distribution in various genomic compartment

Pairs	CDS exon	UTR exon	Intron	Intergenic
**SINE-LINE**	5.17E-51	5.6E-145	0	0
**SINE-LTR**	6E-146	2.37E-14	0	0
**SINE-DNA**	2.51E-24	0.097711	0	0
**LINE-LTR**	1.3E-27	6.17E-54	3.59E-43	2.27E-39
**LINE-DNA**	0.001762	1.98E-28	0	0
**LTR-DNA**	0.005956	0.032449	0	0

We used Fisher's exact test to evaluate significance in various genomic compartment between two TE class pairs. When a region was mapped to different compartments, it followed this priority: CDS exons > UTR exons > Introns > Intergenic regions.

S2 Table: The expression distribution of subfamilies in 80 groups

	Number	Percentage
**non-hub TEs**	265	30.99%
**hub TEs**	131	15.32%
**commonly expressed TEs**	66	7.72%
**zeros expressed TEs**	393	45.96%

Among 855 subfamilies, 393 subfamilies were not expressed in any groups. In order to convenient drawing, we divided remained subfamilies into three classed: commonly expressed TEs, hub TEs and non-hub TEs. Commonly expressed TEs represent expressed TEs in all groups. We used 26 as threshold, then were subdivided other subfamilies into hub or non-hub TEs.

S3 Table: The distribution of specifically expressed TEs in four classes.

Class	Simple expression	Percentage 1 (%)	Percentage 2 (%)
**DNA**	3	2.68	6.52
**LINE**	6	6	13.04
**LTR**	37	6.18	80.43

S4 Table: The distribution of organ-specific TEs in four classes.

Class	organ-specific TEs	Percentage 1 (%)	Percentage 2 (%)
**LTR**	81	13.52	89.01
**LINE**	2	2	2.20
**DNA**	8	7.14	8.79

S5 Table: The number of development-dependent DETSs in all organs.

Organ	21_100	6_100	2_100	6_21	2_21	2_6	Unique
**Ad**	0	1	7	0	7	8	9
**Br**	0	0	0	0	0	0	0
**He**	1	0	1	0	0	0	1
**Ki**	0	0	5	0	6	5	8
**Li**	1	3	4	1	5	2	7
**Lu**	0	0	2	0	0	0	2
**Mu**	0	1	2	0	1	0	2
**Sp**	1	0	0	0	0	1	2
**Te**	44	47	16	0	43	44	65
**Th**	0	4	3	0	0	0	5
**Ut**	0	1	3	0	3	2	4

We used FC $\geqslant 2$ or $\leqslant 0.5$ plus Bonferroni-corrected P-value $\leqslant 0.05$ to identify development-dependent DETSs. The second column to the seventh column shows the number of DETSs between any two developmental stages for the organ. The total number of DETSs is shown in the last column for each organ.

S6 Table: The number of sex-dependent DETEs in different ages

Age	Class	Number	Percentage (%)
**104**	DNA	1	3.85
**21**	DNA	2	7.69
**6**	DNA	1	3.85
**104**	LTR	2	7.69
**21**	LTR	7	26.92
**6**	LTR	12	46.15
**2**	LTR	1	3.85

The third column shows the number about sex-dependent DETEs in specified age and class. The last column shows the proportion in all sex-dependent DETEs.

S7 Table: The information of commonly expressed TEs, including class, family and subfamily.

Subfamily	Class	Family	Subfamily	Class	Family
Alu	SINE	Alu	MER104	DNA	Tc2
AluF_3	SINE	Alu	MER105	DNA	DNA
AluG_3	SINE	Alu	MER112	DNA	MER1_type
Alu_5	SINE	Alu	MER20	DNA	MER1_type
B1F	SINE	Alu	MER3	DNA	MER1_type
B1F1	SINE	Alu	MER34B-int	LTR	ERV1
B1F2	SINE	Alu	MER44C	DNA	MER2_type
B1_Mur1	SINE	Alu	MER46C	DNA	MER2_type
B1_Mur2	SINE	Alu	MER49	LTR	ERV1
B1_Mur3	SINE	Alu	MER50	LTR	ERV1
B1_Mur4	SINE	Alu	MER50B	LTR	ERV1
B1_Rn	SINE	Alu	MER51-int	LTR	ERV1
B3	SINE	B2	MER58	DNA	MER1_type
Charlie8	DNA	MER1_type	MER58C	DNA	MER1_type
FAM	SINE	Alu	MER5A	DNA	MER1_type
FLAM_A	SINE	Alu	MER5B	DNA	MER1_type
FordPrefect	DNA	Tip100	MER66B	LTR	ERV1
ID	SINE	ID	MER66C	LTR	ERV1
ID2	SINE	ID	MER68	LTR	ERVL
ID4	SINE	ID	MER91	DNA	Tip100
ID4_	SINE	ID	MER91A	DNA	Tip100
ID_B1	SINE	B4	MIR	SINE	MIR
L3	LINE	CR1	MIR3	SINE	MIR
L3_Mars	LINE	CR1	MIR_Mars	SINE	MIR
L3b	LINE	CR1	MIRb	SINE	MIR
L4	LINE	RTE	MIRm	SINE	MIR
LTR36	LTR	ERV1	PB1	SINE	Alu
LTR39	LTR	ERV1	PB1D10	SINE	Alu
LTR64	LTR	ERV1	PB1D7	SINE	Alu
LTR9	LTR	ERV1	PB1D9	SINE	Alu
MADE2	DNA	Mariner	RSINE1	SINE	B4
MARNA	DNA	Mariner	THER1_MD	SINE	MIR
MER103	DNA	DNA	Tigger2	DNA	MER2_type

S8 Table : The information of specifically expressed TEs.

Sex	Class	Family	Subfamily	Sex	Class	Family	Subfamily
M	DNA	Tc2	Kanga1a	F	LTR	ERVK	RLTR18
F	LINE	L1	L1M3c	F	LTR	ERVK	RLTR21
M	LINE	L1	L1M3d	M	LTR	ERVK	RLTR43B
F	LINE	L1	L1M5	M	LTR	ERVL	RMER15-int
M	LINE	L1	L1MCc	M	LTR	ERVK	RMER17D2
F	LINE	L1	L1MD1	F	LTR	ERVK	RMER3B-int
M	LINE	L1	L1MEb	M	LTR	ERVK	RMER3D2
M	LTR	ERVL	LTR16B	M	LTR	ERVK	RMER6A
F	LTR	ERV1	LTR37A	F	LTR	ERVK	RNERVK22
M	LTR	ERV1	LTR37B	M	LTR	ERV1	RNLTR10A-int
F	LTR	ERV1	LTR73	M	LTR	ERVK	RNLTR11b-int
M	DNA	Tip100	MER69B	F	LTR	ERVK	RNLTR14-int
M	LTR	ERVL	MER74C	F	LTR	ERV1	RNLTR21
F	LTR	ERVL	MER77	M	LTR	ERV1	RNLTR21-int
M	DNA	DNA	MER99	M	LTR	ERVK	RNLTR3c-int
F	LTR	MaLR	MLT1D	M	LTR	ERV1	RNLTR5A
F	LTR	MaLR	MLT1G	M	LTR	ERV1	RNLTR7-int
F	LTR	ERVL	MLT2C2	F	LTR	ERVK	RNLTR9-int
M	LTR	MaLR	ORR1D-int	F	LTR	ERV1	LTR44
M	LTR	MaLR	ORR1D1	F	LTR	MaLR	MLT1F1-int
M	LTR	MaLR	ORR1D2-int	M	LTR	MaLR	MLT1G-int
F	LTR	ERVK	RLTR12A	M	LTR	ERVL	MLT2D-int
M	LTR	ERVK	RLTR15	M	LTR	ERV1	RNLTR5-int-int

S9 Table: The information of organ-specific TEs, including class, subfamily and organ. If a subfamily was only expressed in an organ, this subfamily was defined as organ-specific TEs.

Class	Subfamily	Organ	Class	Subfamily	Organ
LTR	NICER_Rn-int	Uterus	LTR	MERVL-B1	Lung
LTR	MER88	Uterus	LTR	MER67A	Lung
LTR	MER74B	Uterus	LTR	RNLTR5B	Liver
LTR	MER57D-int	Uterus	LTR	RLTR18B-int	Liver
LTR	LTR67	Uterus	LTR	MER83B-int	Liver
LTR	MER31-int	Thymus	LTR	MER76	Liver
LTR	SRV_RN-int	Testis	LTR	MER110-int	Liver
LTR	RNLTR8B	Testis	LTR	RNLTR12	Kidney
LTR	RNLTR3-int	Testis	LTR	MER65D	Kidney
LTR	RNLTR19A	Testis	LTR	MER21B-int	Kidney
LTR	RNLTR19-int	Testis	LTR	MER110	Kidney
LTR	RNLTR18-int	Testis	LTR	BGLII_A	Kidney
LTR	RNLTR18	Testis	LTR	RMER17B-int	Brain
LTR	RNLTR17-int	Testis	LTR	RMER13A-int	Brain
LTR	RNLTR13	Testis	LTR	RLTR26-int	Brain
LTR	RNIAP1aLTR-int	Testis	LTR	MLT1J1	Brain
LTR	RNERVK8d	Testis	LTR	MLT1I-int	Brain
LTR	RMER4B-int	Testis	LTR	MLT1H2-int	Brain
LTR	RMER21A	Testis	LTR	MLT1G1-int	Brain
LTR	RLTR1_Rat2	Testis	LTR	MLT1E2	Brain
LTR	RLTR10-int	Testis	LTR	MLT1E1A-int	Brain
LTR	ORR1D1-int	Testis	LTR	MLT1E1	Brain
LTR	NICER19A-int	Testis	LTR	MLT1A	Brain
LTR	MYSERV_Rn	Testis	LTR	LTR69	Brain
LTR	MT2_Rat1	Testis	LTR	LTR34	Brain
LTR	MT2B-int	Testis	LTR	LTR23-int	Brain
LTR	MLT2F	Testis	LTR	RNLTR7-int-int	Adrenal
LTR	MLT1E	Testis	LTR	RNLTR5-int	Adrenal
LTR	MLT1A0-int	Testis	LTR	RNLTR2b-int	Adrenal
LTR	MER90a	Testis	LTR	RNLTR2a-int	Adrenal
LTR	MER67D-int	Testis	LTR	RNLTR15A2	Adrenal
LTR	MER4B-int	Testis	LTR	RLTR25B-int	Adrenal
LTR	MER34-int	Testis	LTR	MERVL-A	Adrenal
LTR	LTR68	Testis	LTR	MER74A-int	Adrenal
LTR	ERVL-C2	Testis	LTR	LTR48	Adrenal
LTR	ERVL-B3	Testis	LINE	L1ME3	Kidney
LTR	BGLII_B-int	Testis	LINE	L1MA5	Kidney
LTR	RLTR30-int	Spleen	DNA	MER44D	Uterus
LTR	RLTR22_Rat2-int	Spleen	DNA	MER115	Uterus

Class	Subfamily	Organ	Class	Subfamily	Organ
LTR	MER65-int	Spleen	DNA	Arthur1	Muscle
LTR	ERVL-B	Spleen	DNA	Charlie4a	Lung
LTR	MER74A	Muscle	DNA	MER97c	Kidney
LTR	MER73-int	Muscle	DNA	Kanga1c	Kidney
LTR	HERVL40	Muscle	DNA	MER63D	Heart
LTR	NICER2_Rn	Lung	DNA	MER45	Brain
LTR	MLT1H2	Lung			

S10 Table: The information of organ-enriched TE subfamilies.

Organ	Subfamily	Family	Class
Ki	MER97b	MER1_type	DNA
Li	MER83B-int	ERV1	LTR
Ad	LTR58	ERV1	LTR
Br	HERVL-B2	ERVL	LTR
Br	LTR36	ERV1	LTR
Br	MLT1E1A-int	MaLR	LTR
Br	MLT1G1-int	MaLR	LTR
He	MER66A	ERV1	LTR
Mu	MER73-int	ERVL	LTR
Sp	MER66B	ERV1	LTR

S11 Table: The information of sex-dependent DETEs.

Subfamily	Organ	Age	Class	Subfamily	Organ	Age	Class
Ricksha_b	Ad	100	DNA	RNLTR8B-int	Li	21	LTR
MER97a	Ki	21	DNA	RatERVL-B1	Li	21	LTR
RNLTR12-int-int	Ki	21	LTR	MLT2B2-int	Li	2	LTR
MER21B-int	Ki	6	LTR	MER110-int	Li	6	LTR
RMER17D2-int	Ki	6	LTR	MER65D-int	Li	6	LTR
RNLTR12	Ki	6	LTR	RNLTR11b	Li	6	LTR
RNLTR12-int	Ki	6	LTR	RNLTR5C-int	Li	6	LTR
RNLTR12-int-int	Ki	6	LTR	MLT2B2-int	Lu	6	LTR
MLT2D-int	Li	100	LTR	Ricksha_a	Mu	21	DNA
MER110-int	Li	21	LTR	RLTR22_Rat2-int	Sp	100	LTR
RNLTR11b	Li	21	LTR	MLT2B2-int	Sp	6	LTR
RNLTR5C-int	Li	21	LTR	MLT2B2-int	Th	6	LTR
RNLTR7-int	Li	21	LTR	Ricksha_a	Th	6	DNA
