# Supplementary figures and images for: Expression dynamics and relations with nearby genes of rat transposable elements across 11 organs, 4 developmental stages and both sexes
Source: BMC Genomics. 2017 Aug 29;18:666. doi: 10.1186/s12864-017-4078-7 (PMC5576108; doi:10.1186/s12864-017-4078-7)

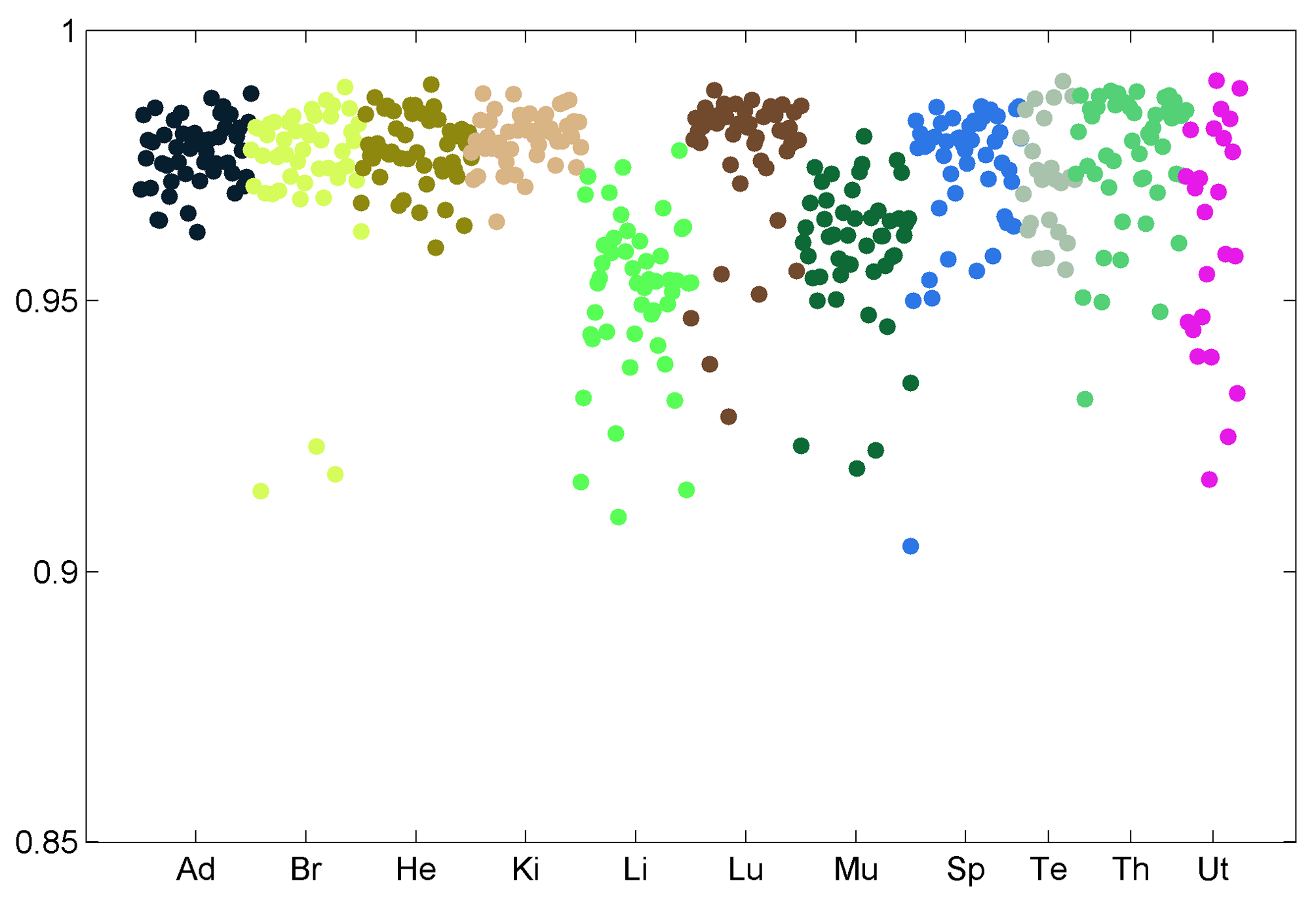

Supplement: Supplementary file 2 — The distribution of pair-wise Pearson correlation coefficient (PCC) in each organ. X-axis represents 480 values in 11 organs, and Y-axis represents PCC between any two of the four biological replicates. Each color represents a kind of organ. Except for Te and Ut, other organs contain 48 dots. (TIFF 298 kb) [file 12864_2017_4078_MOESM2_ESM.tif]

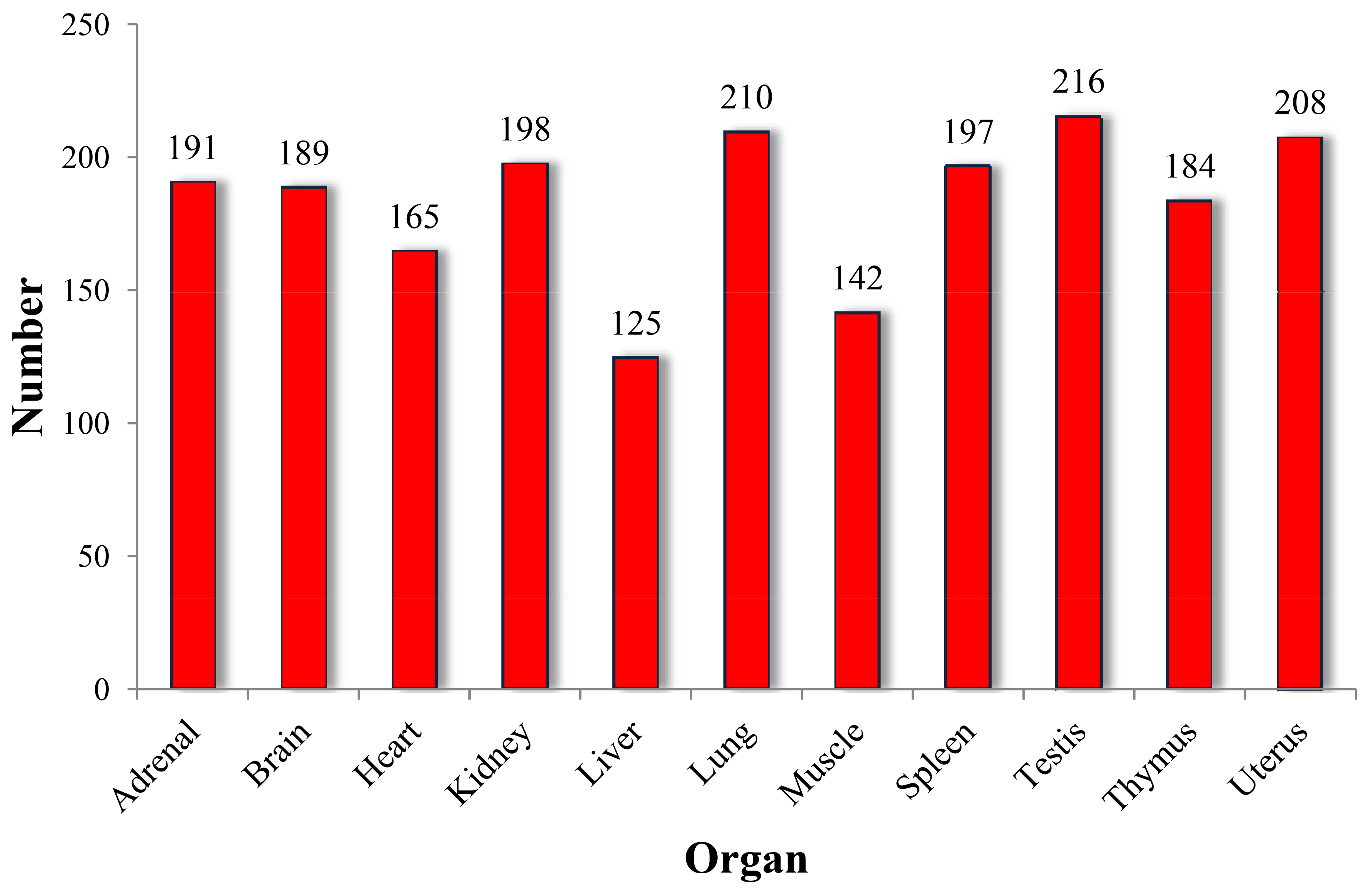

Supplement: Supplementary file 3 — The number of expressed TEs across 11 organs. The x-axis indicates organs and the y-axis indicates the number of expressed TEs. (TIFF 963 kb) [file 12864_2017_4078_MOESM3_ESM.tif]

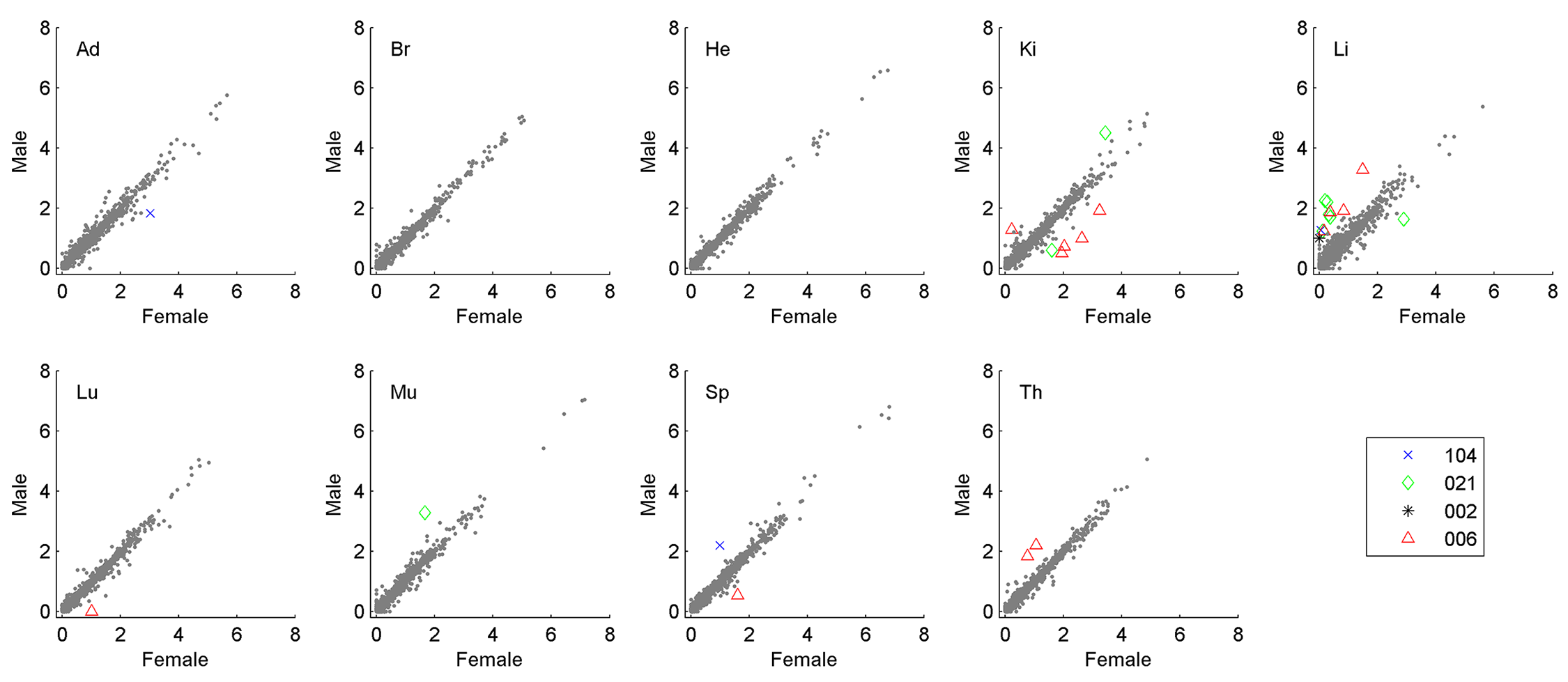

Supplement: Supplementary file 4 — Sex-specific DETEs. Nine organs from four developmental stages. All expression signals are depicted in the scatter plots. X-axis represents expression signal of female rats, and Y-axis represents expression signal of male rats. Non sex-specific TEs are grey color, while DETEs are colored. (TIFF 232 kb) [file 12864_2017_4078_MOESM4_ESM.tif]

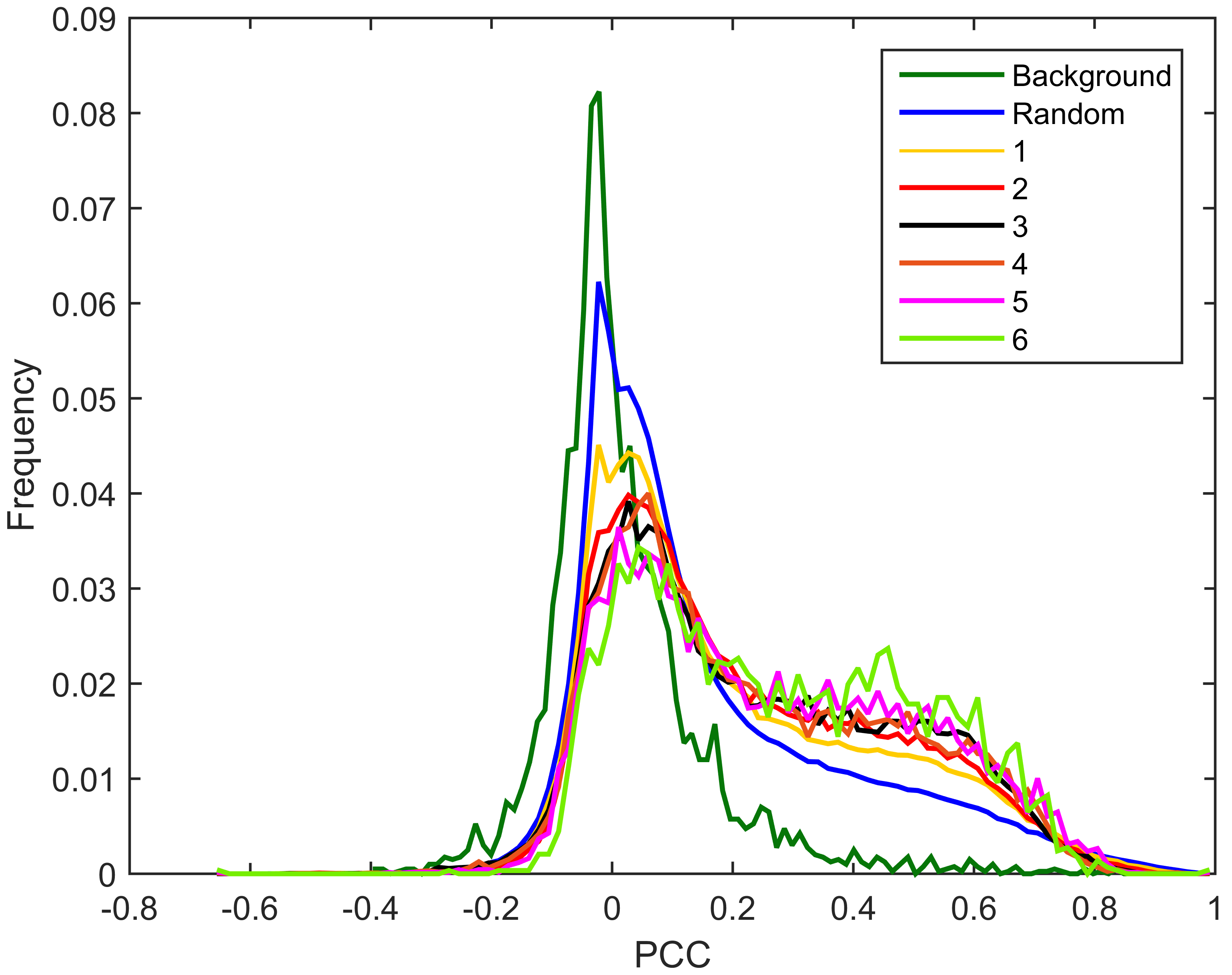

Supplement: Supplementary file 5 — The methods for gene-TE pairs selection. We got all gene-TE pairs by distance. In all genes, some genes were organ-enriched. In all TEs, some TEs were located in UTSSs, while some TEs located in DTSSs. We randomly selected genes and TEs, and these gene-TE pairs were used as Background. We randomly selected gene-TE pairs from all gene-TE pairs that were used as Random. TEs located in UTSSs and organ-enriched genes were combined as Upstream. TEs located in DTSSs and organ-enriched genes were combined as Downstream. (TIFF 639 kb) [file 12864_2017_4078_MOESM5_ESM.tif]

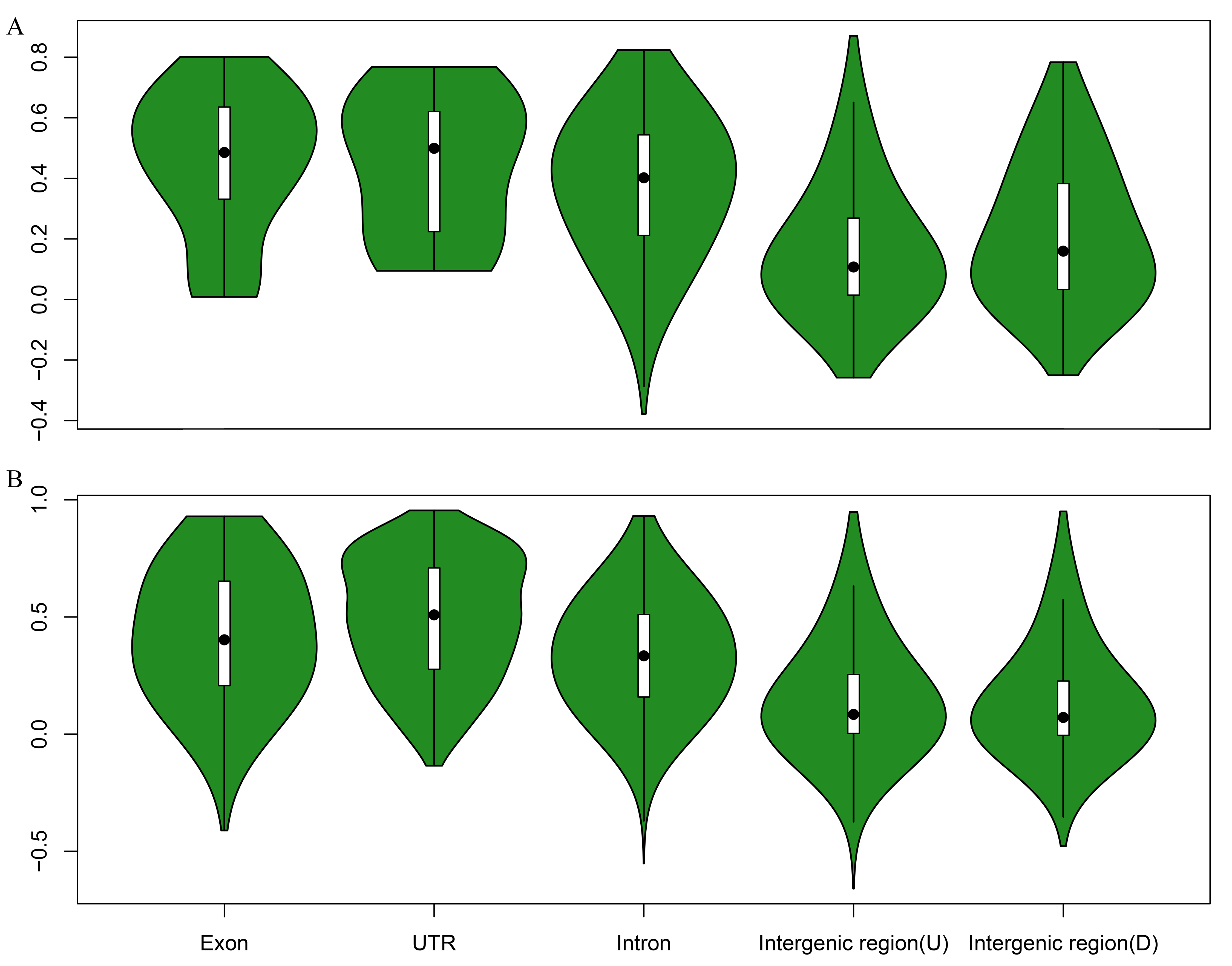

Supplement: Supplementary file 6 — Frequency distribution map of PCC between TEs and sex-specific genes. (TIFF 384 kb) [file 12864_2017_4078_MOESM6_ESM.tif]

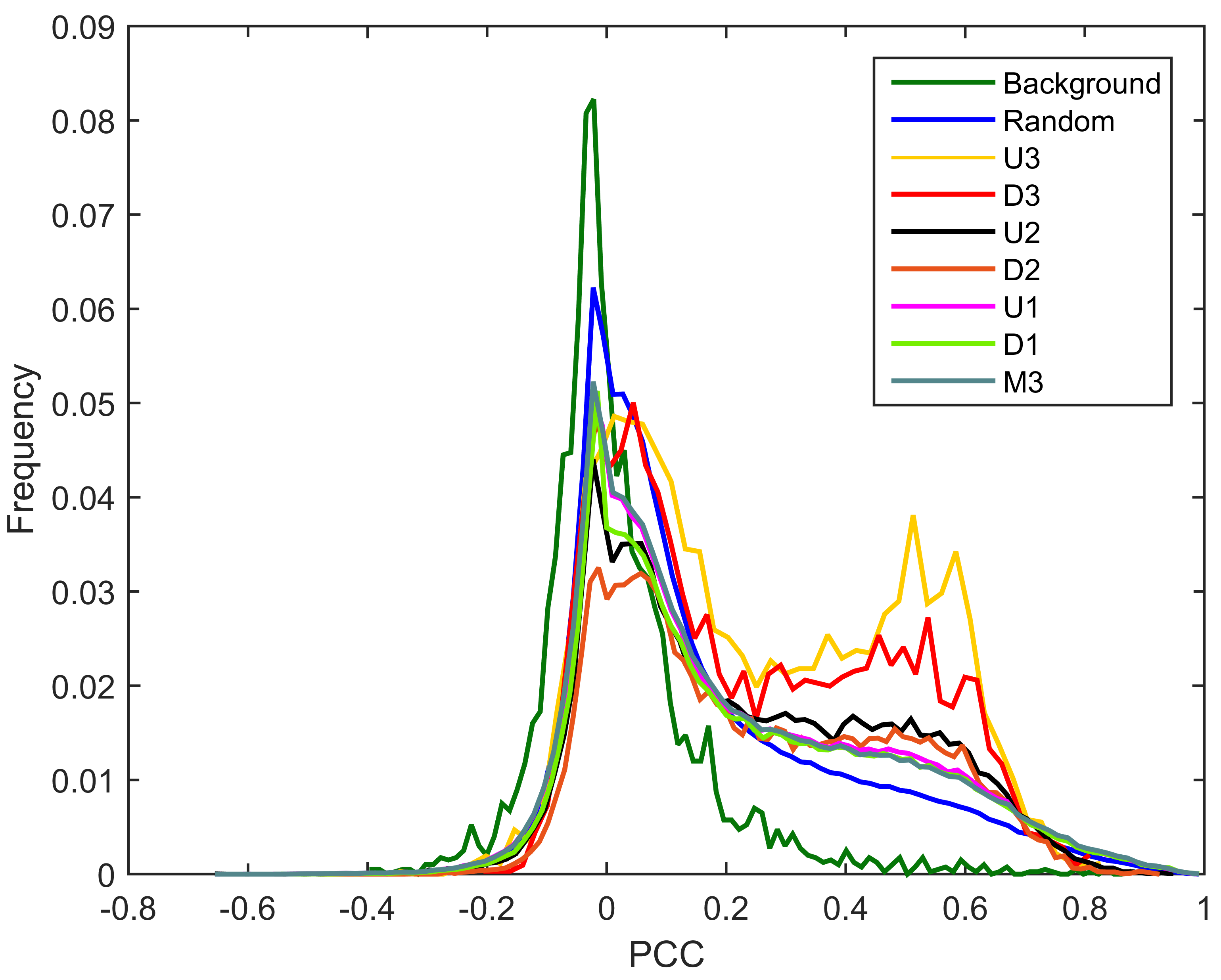

Supplement: Supplementary file 7 — Violin plot of PCC between TE and sex-specific gene that appeared 5 times (A) and 1 time (B) in different organs and development stages. (TIFF 923 kb) [file 12864_2017_4078_MOESM7_ESM.tif]

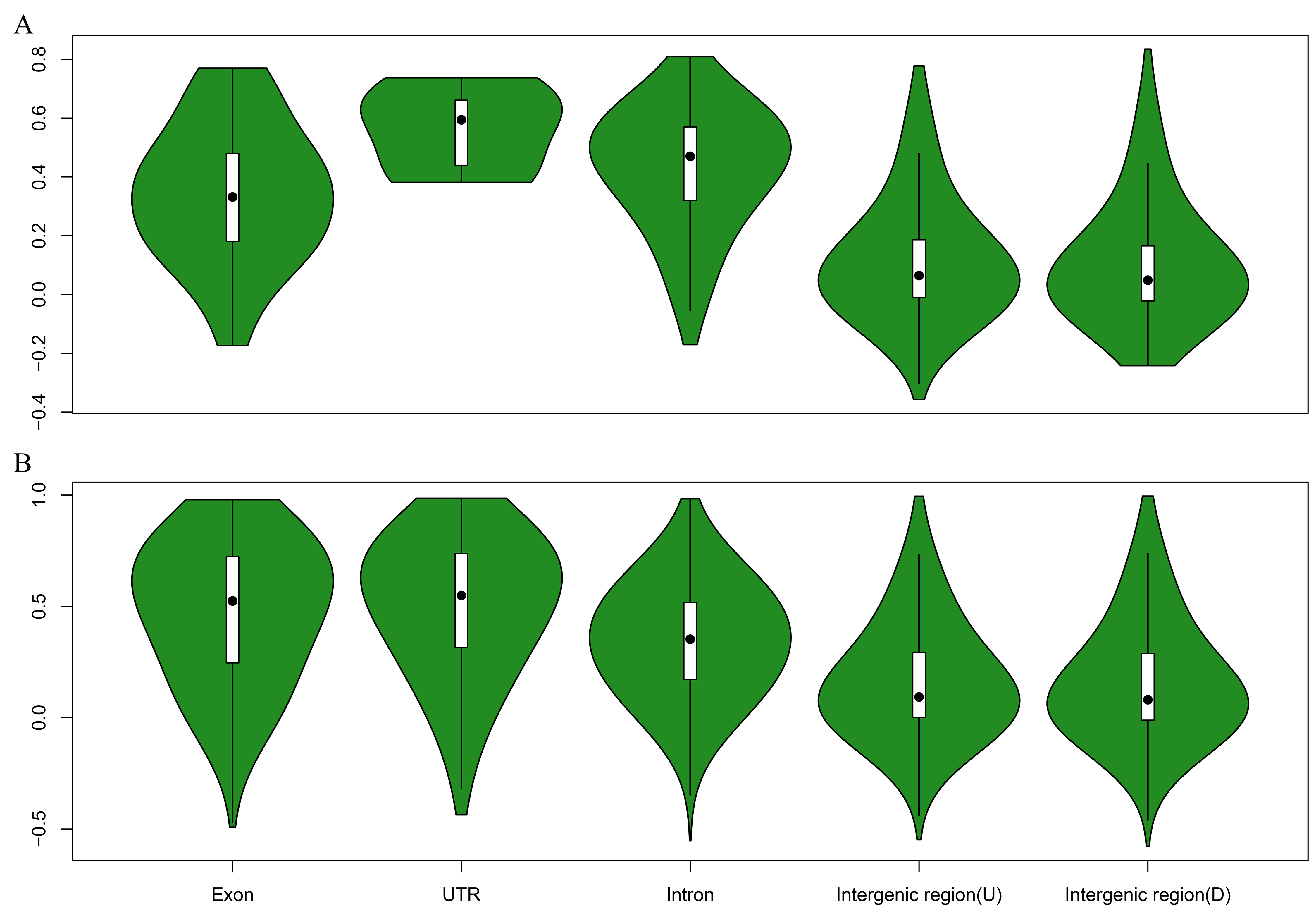

Supplement: Supplementary file 8 — Frequency distribution map of PCC between TEs and development-dependent genes. (TIFF 418 kb) [file 12864_2017_4078_MOESM8_ESM.tif]

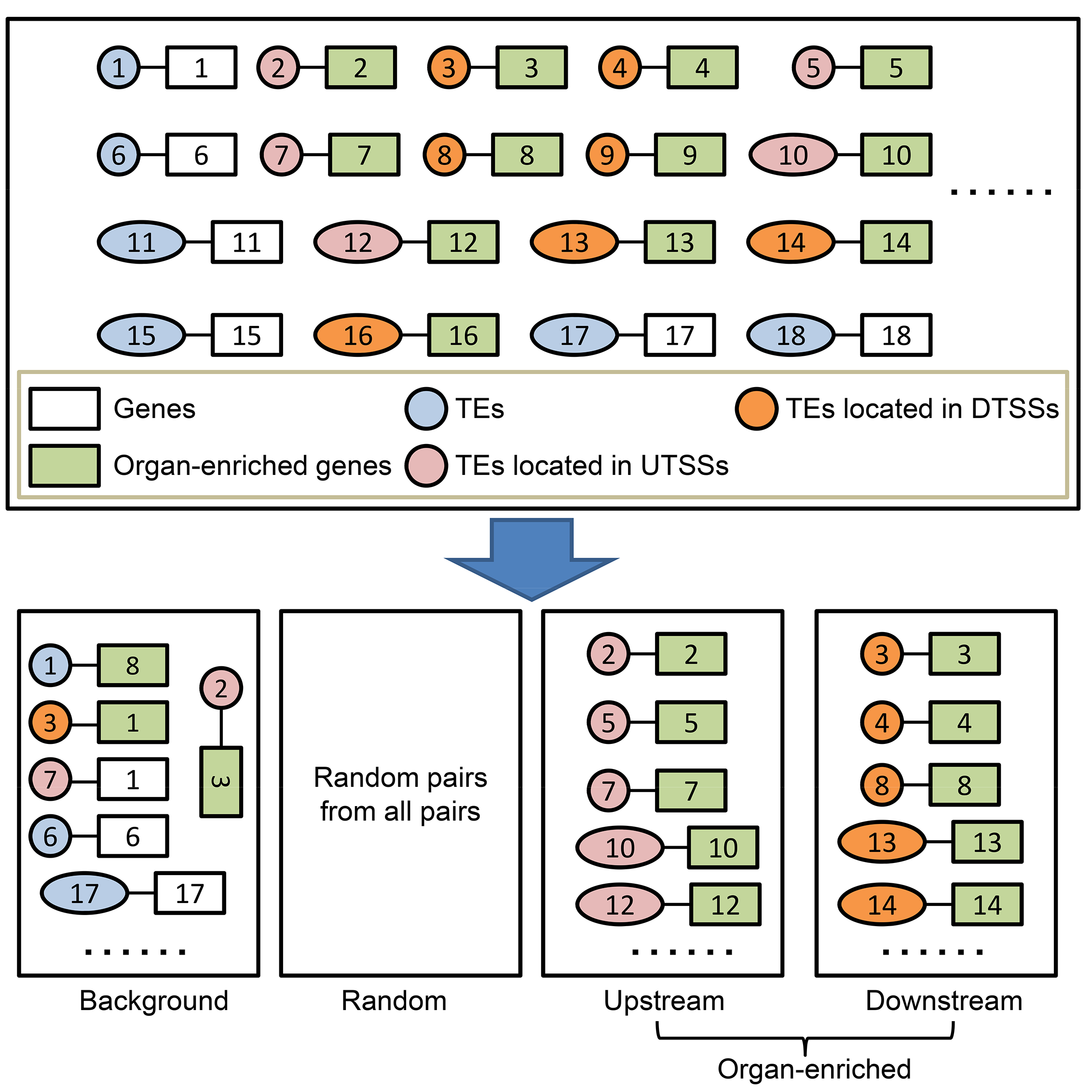

Supplement: Supplementary file 9 — Violin plot of PCC between TE and development-dependent gene that appeared 3 times U (A) and 1 time D (B). (TIFF 797 kb) [file 12864_2017_4078_MOESM9_ESM.tif]
